# The Epidemic

**Published:** 1833

**Authors:** 


### II. The Epidemic.

The reappearance and desolating spread of the Malignant Cholera, has induced us to give up a large portion of this, as of some former numbers, to that subject. The eclectic review which fills an entire department of this number, will give to our remote and insulated readers, whom but few of the treatises on Cholera, ever reach, an adequate idea of the methods of cure which have been found most beneficial elsewhere; and taken, in connexion with what we have previously published, will, indeed, constitute nearly all that has been proposed for that purpose.

That the best of these foreign methods, will be found well adapted to the cure of the Epidemic, in the valley of the Mississippi, is extremely doubtful; for while, its great features remain unaltered, it can scarcely be supposed, that it does not undergo some modification in countries and climates and states of society, so remote from the region where it was first developed. These modifications will, of course, require a modified treatment, and in forming this, will the skill of our Western friends display itself.

We shall be happy to make our Journal the medium, by which they will favor the public with the results of their experience; hoping they will constantly bear in mind, that their papers will be read and esteemed—*cœteris paribus*—in proportion as they are composed of facts and suggestions which have not hitherto been noted.

For a history of the existing Epidemic visitation on this city, we refer to the close of the review to which we have just alluded.

